# Structural implications of hERG K^+^ channel block by a high-affinity minimally structured blocker

**DOI:** 10.1074/jbc.RA117.000363

**Published:** 2018-03-15

**Authors:** Matthew V. Helliwell, Yihong Zhang, Aziza El Harchi, Chunyun Du, Jules C. Hancox, Christopher E. Dempsey

**Affiliations:** From the Schools of ‡ Biochemistry and; § Physiology, Pharmacology, and Neuroscience, University of Bristol, Bristol BS8 1TD, United Kingdom

**Keywords:** hERG, heart, drug action, molecular pharmacology, potassium channel, mutagenesis, molecular docking, alanine scan mutagenesis, long QT syndrome

## Abstract

Cardiac potassium channels encoded by human ether-à-go-go–related gene (*hERG*) are major targets for structurally diverse drugs associated with acquired long QT syndrome. This study characterized hERG channel inhibition by a minimally structured high-affinity hERG inhibitor, Cavalli-2, composed of three phenyl groups linked by polymethylene spacers around a central amino group, chosen to probe the spatial arrangement of side chain groups in the high-affinity drug-binding site of the hERG pore. hERG current (*I*_hERG_) recorded at physiological temperature from HEK293 cells was inhibited with an IC_50_ of 35.6 nm with time and voltage dependence characteristic of blockade contingent upon channel gating. Potency of Cavalli-2 action was markedly reduced for attenuated inactivation mutants located near (S620T; 54-fold) and remote from (N588K; 15-fold) the channel pore. The S6 Y652A and F656A mutations decreased inhibitory potency 17- and 75-fold, respectively, whereas T623A and S624A at the base of the selectivity filter also decreased potency (16- and 7-fold, respectively). The S5 helix F557L mutation decreased potency 10-fold, and both F557L and Y652A mutations eliminated voltage dependence of inhibition. Computational docking using the recent cryo-EM structure of an open channel hERG construct could only partially recapitulate experimental data, and the high dependence of Cavalli-2 block on Phe-656 is not readily explainable in that structure. A small clockwise rotation of the inner (S6) helix of the hERG pore from its configuration in the cryo-EM structure may be required to optimize Phe-656 side chain orientations compatible with high-affinity block.

## Introduction

The human ether-à-go-go–related gene encodes the hERG^4^ potassium (K^+^) channel, which carries the rapid delayed rectifier repolarizing current (*I*_Kr_) in human cardiac myocytes. This current contributes to ventricular action potential repolarization and effectively controls the duration of the QT interval in humans (1–3). The repolarizing properties of hERG are mediated by rapid channel inactivation following channel opening upon membrane depolarization followed by rapid recovery from inactivation and slow channel closing (deactivation) at repolarizing membrane potentials (1, 2). hERG continues to be of intense pharmacological interest due to the wide variety of cardiac and noncardiac drugs that block the channel with the potential to cause acquired long QT syndrome (aLQTS) and *torsades de pointes (TdP)* arrhythmia, which can lead to sudden cardiac death (3, 4). Accordingly, new pharmaceutical compounds must undergo electrophysiological screening for hERG/*I*_Kr_ block early during drug development (3, 5).

**Figure 1.**
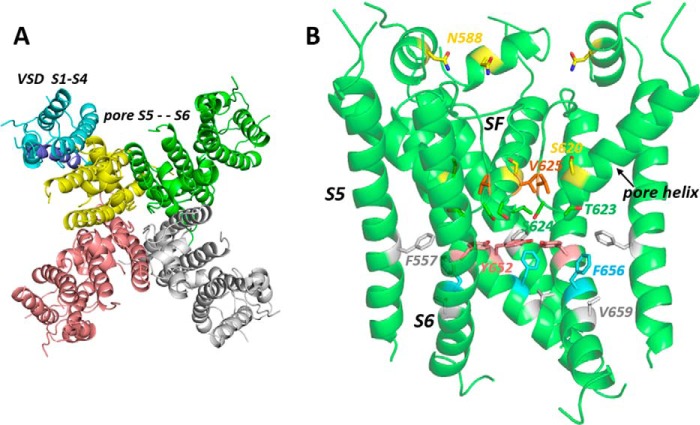
**Open pore cryo-EM structure of a hERG construct from Wang and MacKinnon (8).** *A* is a top-down view illustrating the subunit arrangement of the hERG construct membrane domain tetramer. The voltage sensor domain (*VSD*) (transmembrane helices S1–S4) of one subunit is colored *blue. B*, side view of the pore domain comprising the S5 helix, pore helix, selectivity filter (*SF*), and S6 helix; the extracellular turret linking the top of S5 and the N-terminal end of the pore helix has some missing atom density. Amino acid residues mutated in this study or otherwise described in the text are highlighted.

A substantial body of research has established that the majority of hERG-blocking drugs bind within the channel pore that comprises the S5 and S6 helices and a connecting loop that lies in or near the extracytoplasmic membrane surface (2, 4, 6, 7). The latter sequence contains a long extracellular domain that is involved in rapid C-type inactivation at moderate depolarized potentials, a pore helix, and a canonical selectivity filter sequence (see Fig. 1 and Ref. 8). The hERG pore is a tetramer (4 × S5-S6), and each monomer is connected by a short S4-S5 linker to separate four transmembrane helix voltage sensor domains (transmembrane helices S1–S4) that couple gating of the K^+^-conducting pore to changes in the membrane potential (see Fig. 1). Because channel block by almost all hERG-blocking drugs is attenuated by mutations of one or both of two key residues (Tyr-652 and Phe-656) on the S6 helix that forms much of the lining of the K^+^ conductance pathway, these drugs, particularly the large number that contain a positively charged amino group, are proposed to bind within the K^+^ conduction pathway at some point below the bottom of the selectivity filter (see Fig. 1). Many positively charged hERG blockers access this region of the channel pore when the channel is gated open upon membrane depolarization.

Alanine-scanning mutagenesis has been used to further define the binding determinants for several hERG blockers, including MK-499 (9), propafenone (10), vesnarinone (11), cisapride (12), terfenadine (12), quinidine (13), chloroquine (14), dofetilide (15), E-4031 (15), ibutilide (16, 17), clofilium (16, 17), disopyramide (18), ranolazine (19), lidocaine (19), and amiodarone (20). In general, a selection of residues that line the hERG conduction pathway (Thr-623, Ser-624 at the bottom of the pore helix, and Tyr-652 and Phe-656 on S6) is important for drug block; the wide variation in the structures of hERG blockers is proposed to arise from the large set of residues (*e.g.* eight Tyr-652 and Phe-656 aromatic side chains in total) available for drug interactions. Attenuation of channel inactivation in N558K (21) and especially S620T hERG mutants (22) (see Fig. 1 for location of these residues) is associated with partial (N588K) or stronger (S620T) attenuation of hERG block by high-affinity blockers, indicating that retention of inactivation is necessary for block. Interestingly, the noninactivating EAG channels, in the same channel family as hERG, possess aromatic residues in positions equivalent to Tyr-652 and Phe-656 but do not exhibit the same susceptibility to drug block (22, 23). Inactivation is thought to involve conformational changes that alter the configurations of side chains that interact with drugs in the pore cavity (24). Whether this indicates that these blockers bind more strongly to the inactivated state has not been established.

Until very recently a structure for the hERG membrane domain was unavailable, and homology models of the hERG pore have been used to distinguish residues that may interact directly with hERG-blocking drugs and those whose mutation to alanine reduces drug block by nondirect, allosteric effects (6, 10, 25–29). The latter side chains include those of Val-625, which lies behind the K^+^ selectivity filter and is almost certainly inaccessible to direct interaction with blockers in the channel pore, and Val-659, which likely lies on the side of the S6 helix directed away from the K^+^ permeation pathway (see Fig. 1). Attenuation of channel block in V625A and V659A mutants probably results from the attenuation of inactivation in these mutants in parallel with attenuation of high-affinity block in inactivation-deficient mutants N558K and S620T. The effect of T623A hERG in attenuating drug block might also result from nondirect effects because, in drug docking with some hERG pore models, drug poses that make simultaneous interactions with Tyr-652 and/or Phe-656 as well as Thr-623 are often not found (25, 27, 29).

**Figure 2.**
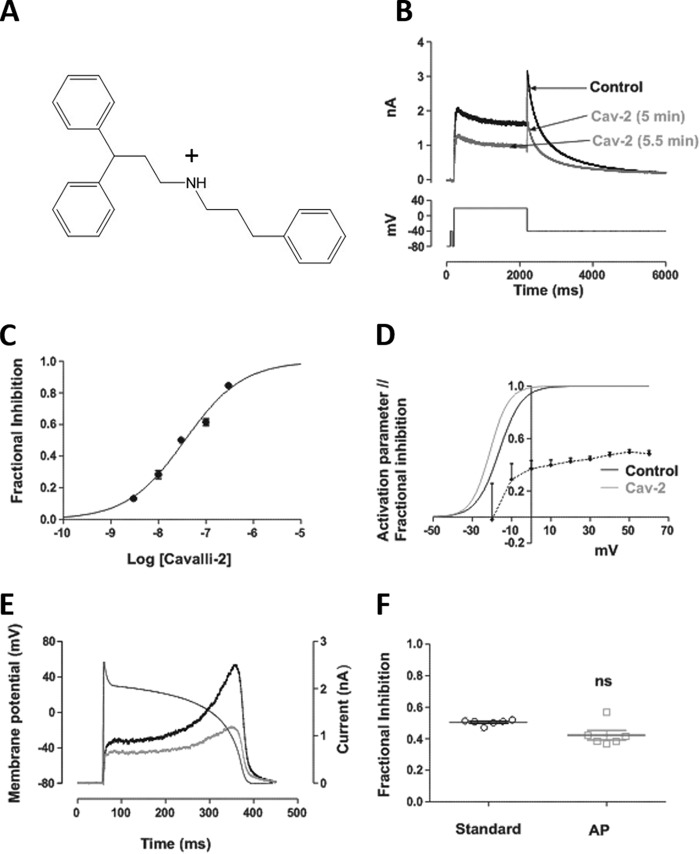
**Effect of Cavalli-2 on WT *I*_hERG_.** *A*, structure of the “minimally structured” compound Cavalli-2. *B*, *upper panel*, shows representative traces recorded in 4 mm normal [K^+^]*_e_* elicited by depolarizing voltage command (*lower panel*) in the absence (*black*) and presence (gray) of 30 nm Cavalli-2 (*Cav-2*) after 5 and 5.5 min to demonstrate steady-state block. *C*, concentration-response relationships for inhibition of *I*_hERG_ tails at −40 mV by Cavalli-2 (*n* ≥ 5 for each point). *D*, voltage dependence of Cavalli-2 block (*black dotted line*) and voltage-dependent activation relationships for *I*_hERG_ in control (*black continuous line*) and in the presence of 30 nm Cavalli-2 (*gray line*) (*n* = 6). *V*_0.5_ = −16.0 ± 3.6 mV and *k* = 5.37 ± 0.75 and *V*_0.5_ = −21.2 ± 2.9 mV and *k* = 5.03 ± 1.50 in control and in the presence of 30 nm Cavalli-2, respectively. *E*, representative *I*_hERG_ records in control (*black*) and in the presence of 30 nm Cavalli-2 (*red line*) elicited by the superimposed action potential waveform. *F*, scatter plot comparing fractional block of *I*_hERG_ by 30 nm Cavalli-2 using the standard protocol and action potential waveform. *n* = 6; *p* < 0.05, unpaired *t* test. *Error bars* represent means ± S.E. *ns*, not significant.

One way to better define hERG pore domain side chains that interact directly with blockers is to use structurally simplified molecules that retain high blocking affinity but can make only a minimal set of interactions. A series of “minimal hERG blockers” was described by Cavalli *et al.* (30) that consist of three phenyl (or fluorophenyl) groups linked by polymethylene spacers around a central amino group. High-affinity analogues of the Cavalli series might be expected to make three aromatic interactions with aromatic side chains in the hERG pore and one “polar” interaction involving the positively charged amino group and so should be useful in assessing the spatial relationships of aromatic side chains within the pore. Here, we describe the blocking effect of one of the Cavalli series (“Cavalli-2”; see Fig. 2*A*) on wildtype (WT) hERG and a series of alanine replacement mutants involving hERG pore residues previously shown to be important for drug block. A recent study reported a significant reduction of drug block in a hERG F557L mutant with a suggestion that this residue on the S5 helix (see Fig. 1) might directly interact with some hERG blockers (28); we have included this mutant together with our alanine scan for Cavalli-2 channel block. The recent high-resolution open pore structure of a hERG construct using cryo-EM (Ref. 8 and see Fig. 1) allows our mutant data on Cavalli-2 block to be interpreted in the context of an open pore structure existing at a neutral (0-mV) membrane potential. The interpretation of interaction of Cavalli-2 with hERG pore residues in high-affinity bound states is also of interest in the context of the strong structural similarity of Cavalli-2 with prenylamine, one of the many drugs removed from the market due to cardiac side effects likely resulting from hERG channel block (31).

## Results

### Concentration dependence of I_hERG_ inhibition by Cavalli-2

Five concentrations of Cavalli-2 on *I*_hERG_ were investigated using the protocol shown in Fig. 2*B*, which has been used in prior studies from our laboratory (18–20, 32–34). From −80 mV, a 2-s activating step to +20 mV was followed by a 6-s step to −40 mV to elicit *I*_hERG_ tails. *I*_hERG_ tail magnitude was measured relative to the brief depolarizing prepulse to −40 mV (18–20, 32–34), and fractional inhibition of *I*_hERG_ was calculated using Equation 1. *I*_hERG_ inhibition developed progressively over 5 min. In the example traces shown in Fig. 2*B*, the *I*_hERG_ tail was inhibited by ∼50% by 30 nm Cavalli-2 with a mean level of inhibition of 50.2 ± 2.0% (*n* = 7). Washout was attempted in five cells with partial recovery to 59.5 ± 4.1% of control amplitude. Fig. 2*C* shows the mean concentration-response relationship for *I*_hERG_ tail inhibition by Cavalli-2; a fit to the data using Equation 2 yielded a half-maximal inhibitory concentration (IC_50_) value of 35.6 nm (confidence interval (CI), 30.9–41.1) with a Hill slope of 0.69 (CI, 0.62–0.77). Voltage dependence of inhibition was assessed using a similar, modified protocol with test depolarizations to voltages between −30 and +60 mV (Fig. S1). In each of control and Cavalli-2, the *I*_hERG_ activation relationship was derived from current-voltage plots of *I*_hERG_ tails, and *V*_0.5_ and *k* values were derived from these using a fit to the data with Equation 3. These values were used to calculate activation variables between −60 and +60 mV. In control, the mean *V*_0.5_ and *k* values were, respectively, −16.0 ± 3.5 mV and 5.4 ± 0.8; in 30 nm Cavalli-2, these values were −21.1 ± 2.9 mV and 5.0 ± 1.5 (*V*_0.5_, *p* < 0.05; slope, not significant, paired *t* test; *n* = 5). Fractional inhibition of *I*_hERG_ was voltage-dependent over the tested range (*p* < 0.05, one-way ANOVA). *I*_hERG_ tail inhibition was steeply dependent on voltage at membrane potentials coinciding with the rising phase of the *I*_hERG_ activation relationship. An increase in *I*_hERG_ was seen at −30 mV in Cavalli-2, which correlated with the leftward shift in voltage-dependent activation. Notably, fractional inhibition also showed some increase over voltages at which the activation relationship had reached a plateau. This feature was used to probe block further using Woodhull analysis (Equation 4 (35, 36)), yielding a δ value of 0.2. In separate experiments, *I*_hERG_ inhibition during a physiological (ventricular action potential (AP)) waveform was assessed using AP voltage clamp (Fig. 2*E*). Fig. 2*F* shows that 30 nm Cavalli-2 inhibited peak *I*_hERG_ during the AP by 42.3 ± 6.7%, which was not significantly different from that with the standard protocol shown in Fig. 2*B* (unpaired *t* test; *n* = 6).

### Development of inhibition during an envelope-of-tails protocol

**Figure 3.**
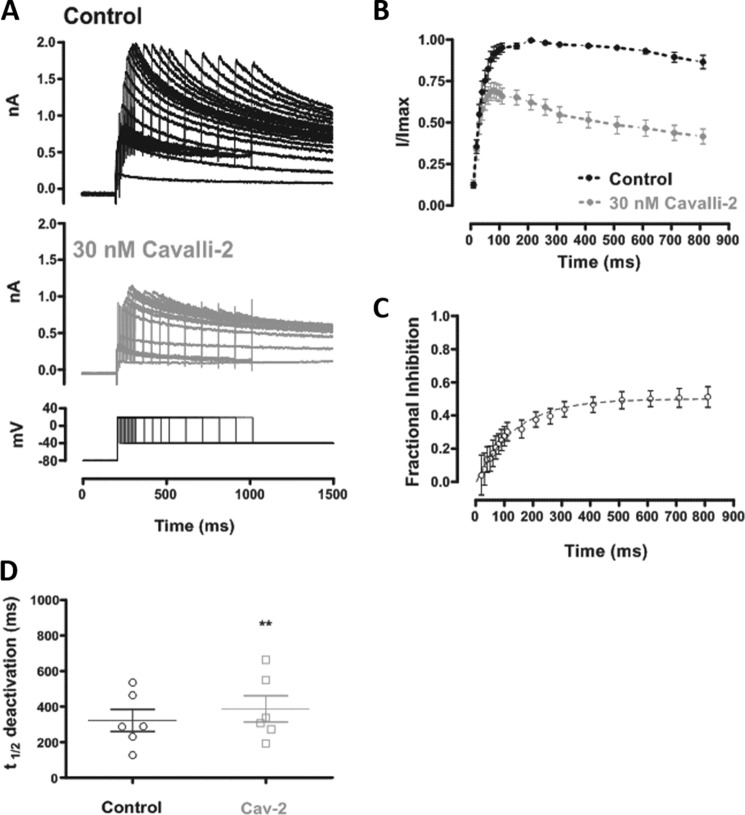
**The time dependence of *I*_hERG_ inhibition by Cavalli-2.** *A*, representative traces of *I*_hERG_ in control (*upper panel*) and in the presence of 30 nm Cavalli-2 (*lower panel*) elicited by the “envelope-of-tails” protocol shown at the *bottom* of the *lower panel. B*, time dependence of normalized tail *I*_hERG_ in control (*black*) and in the presence of 30 nm Cavalli-2 (*gray*) (*n* = 6). Data at each time point were normalized to the maximum tail current elicited by the protocol in control. *Lines* connect successive points in each plot. *C*, time dependence of fractional block of *I*_hERG_ by 30 nm Cavalli-2 fitted with a monoexponential function (*n* = 6; time constant = 140.9 ± 33.4 ms). *D*, scatter plot comparing *t*_½_ of *I*_hERG_ deactivation in control and after application of 30 nm Cavalli-2 (*Cav-2*) using the protocol shown in Fig. 2*B. n* = 6; unpaired *t* test. *Error bars* represent means ± S.E.

The results shown in Fig. 2 were suggestive of strong dependence of *I*_hERG_ inhibition on channel gating. This was investigated further using an envelope-of-tails protocol (34, 37–40), shown in Fig. 3*A*, *lower panel*. The protocol was first applied in control solution and then discontinued while the cell under study was rested in the presence of Cavalli-2 for 5 min. The protocol was then reapplied once in the presence of Cavalli-2, and fractional block of the *I*_hERG_ tail was evaluated for each of the different duration voltage commands. In both control and Cavalli-2, the magnitude of the *I*_hERG_ tail increased progressively with test pulse duration, but control and 30 nm Cavalli-2 traces were similar for very short commands and diverged for longer duration commands as shown in Fig. 3*B*. Fig. 3*C* shows a plot of mean fractional inhibition against pulse duration for this protocol. For the shortest activating pulses, little or no *I*_hERG_ block was seen, and inhibition developed progressively with longer duration pulses. The time course of development of *I*_hERG_ inhibition during the envelope-of-tails protocol was fitted with a single exponential equation, yielding a τ value of 129.1 ± 23.1 ms (*n* = 5).

*I*_hERG_ tails on repolarization to −40 mV elicited by the standard protocol in Fig. 2*B* were used to assess effects of Cavalli-2 on *I*_hERG_ deactivation. Values for *t*_½_ deactivation were calculated from the peak tail current to the end of the repolarizing pulse in control and after application of 30 nm Cavalli-2. No significant change in *t*_½_ deactivation was observed in the presence of Cavalli-2 (unpaired *t* test; *n* = 6).

### Inactivation

**Figure 4.**
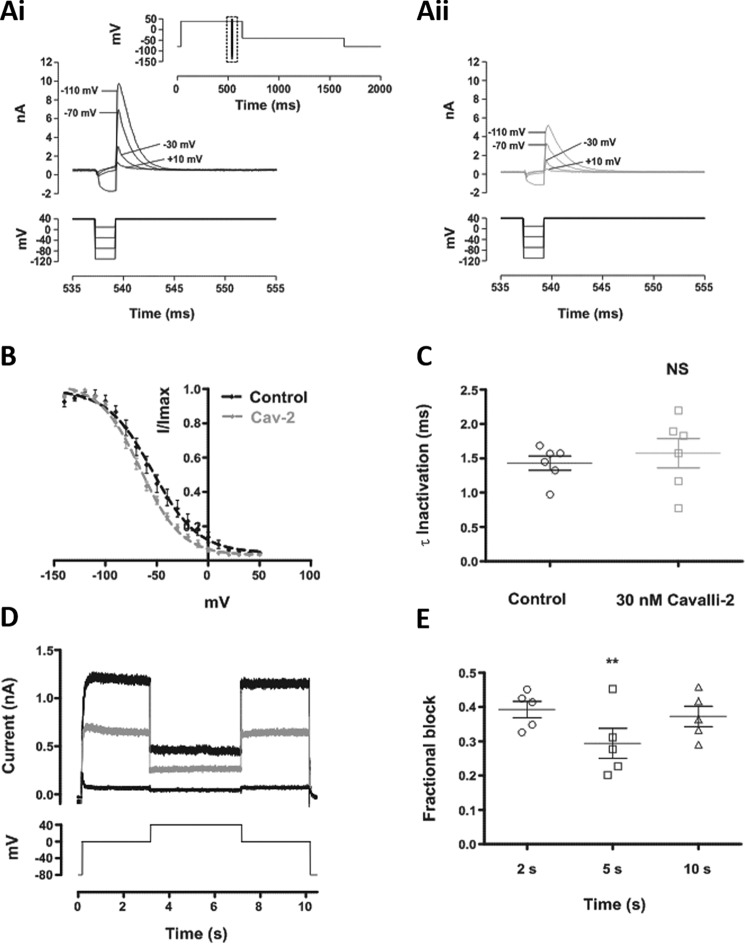
**Effect of Cavalli-2 on hERG channel availability.** *A*, *upper* traces show WT *I*_hERG_ elicited by the “availability protocol” (from a holding potential of −80 mV, the membrane was depolarized to +40 mV (500 ms) and then briefly (2 ms) repolarized to a test potential ranging from −140 to +50 mV before returning to +40 mV). The full protocol is shown in the *inset*; the traces focus on the *boxed* area from the full protocol in control (*A*, *panel i*) and in the presence of 30 nm Cavalli-2 (*A*, *panel ii*). *B*, voltage dependence of the normalized resurgent current elicited by the third step of the availability protocol in control (*black*) and in the presence of 30 nm Cavalli-2 (*Cav-2*) (*gray*) (*n* = 6). *V*_0.5_ = −56.0 ± 1.9 mV and *k* = 21.3 ± 1.9 and *V*_0.5_ = −62.4 ± 1.4 mV and *k* = 20.1 ± 1.3 in control and in the presence of 30 nm Cavalli-2, respectively. *C*, scatter plots comparing time constants of *I*_hERG_ inactivation calculated by fitting the peak transient current at +40 mV after a 2-ms step to −120 mV with a monoexponential decay function (*n* = 6; *NS*, not significant, *p* > 0.05, Wilcoxon matched-pairs signed-rank test). *D*, current records in control (*thick black line*) and after application of 30 nm Cavalli-2 (*gray line*) elicited by the voltage protocol shown (*lower* trace) applied from a holding potential of −80 mV. The *thin black line* shows current remaining after application of 5 μm E-4031. *E*, scatter plot comparing level of *I*_hERG_ block at 2 (0 mV), 5 (+40 mV), and 10 s (0 mV). *n* = 5; **, *p* < 0.005, one-way ANOVA. Scatter plots in *C* and *E* show individual data points. All *error bars* represent means ± S.E.

Taken together, the data in Figs. 2 and 3 indicate that the inhibitory action of Cavalli-2 is strongly contingent upon hERG channel gating. The action of several high-affinity *I*_hERG_ inhibitors is significantly dependent on inactivation gating (21, 22, 41). Several approaches were taken to investigate the role of inactivation gating in the action of Cavalli-2. First, a standard three-step protocol (Fig. 4*A*, *panel i*, *inset*) was used to determine voltage-dependent availability of *I*_hERG_ in control conditions and in the presence of Cavalli-2. Fig. 4*B* shows mean plots of *I*_hERG_ availability, fitted with Equation 5. The *V*_0.5_ of *I*_hERG_ inactivation in control was −54.2 ± 2.2 mV, and *k* was 22.1, whereas in 30 nm Cavalli-2, the *V*_0.5_ was −65.8 ± 1.1, and *k* was 19.9 ± 1.1. Thus, Cavalli-2 caused a modest but statistically significant leftward shift in voltage-dependent inactivation (*p* = 0.0005; paired *t* test; *n* = 6), consistent with stabilization of the inactivated state by the drug. *I*_hERG_ currents elicited by the third step following a brief repolarizing step to −120 mV were used to determine the time course of inactivation. The inactivation τ (obtained from Equation 6) in control was 1.4 ± 0.2 ms, whereas in Cavalli-2 it was 1.6 ± 0.5 ms (*n* = 6; *p* > 0.05; Wilcoxon matched-pairs signed-rank test). Thus, Cavalli-2 did not statistically significantly alter the time course of development of inactivation.

Inactivation dependence of the compound's effect on WT *I*_hERG_ was investigated using the three-step protocol shown in Fig. 4*D*. Membrane potential was stepped from −80 to 0 mV for 3 s after which a further 4-s step to +40 mV to promote *I*_hERG_ inactivation was applied before repolarization back to 0 mV for 3 s prior to returning membrane potential to −80 mV. The protocol was applied first in control solution and then following a 5-min exposure to Cavalli-2 at −80 mV in the absence of pulsing. In both conditions, outward current developed during the initial 0-mV command that was reduced during the +40-mV step, reflecting enhanced inactivation during that phase of the protocol. A supramaximal concentration (5 μm) of E-4031 was used to enable subtraction of background current at each phase of the protocol, and then fractional inhibition of *I*_hERG_ was compared during the different phases of voltage command. Fig. 4*E* shows that fractional inhibition of *I*_hERG_ by Cavalli-2 was significantly reduced during the +40-mV phase (*n* = 5; *p* < 0.005, one-way ANOVA). This is consistent with a slightly stronger affinity of the compound for activated than inactivated channels.

**Figure 5.**
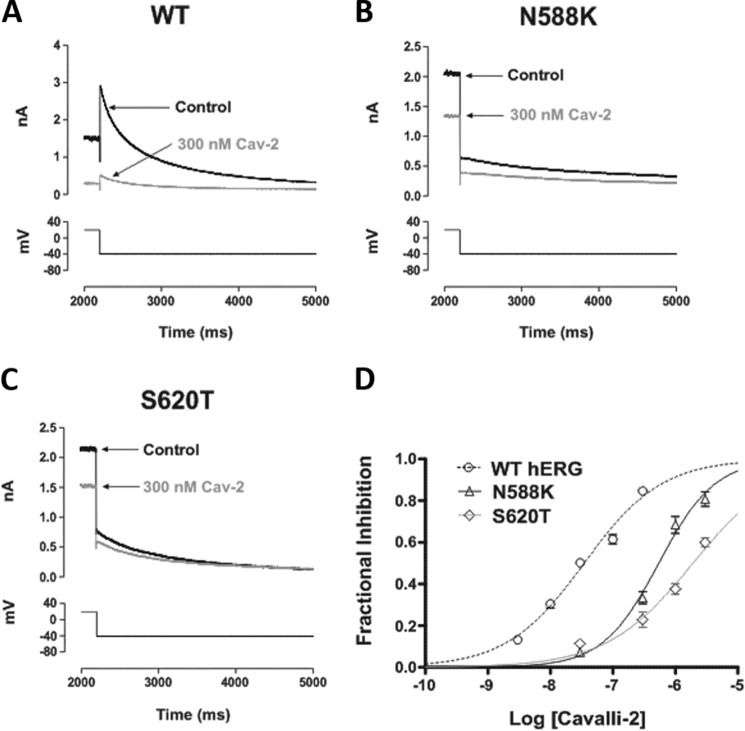
**Cavalli-2 blockade of *I*_hERG_ carried by inactivation-attenuated mutants.** Representative current traces from inactivation-attenuated mutants (*B*, N588K; *C*, S620T, which is more profoundly inactivation-deficient than N588K) in the absence and presence of 300 nm Cavalli-2 (*Cav-2*) using the voltage protocol described in Fig. 1 are shown. WT *I*_hERG_ traces in control and after application of 300 nm Cavalli-2 are shown in *A* as a comparator. *D*, concentration-response relationships for inhibition of N588K and S620T *I*_hERG_ tails at −40 mV by Cavalli-2. *n* ≥ 5 for each concentration of each curve. *Error bars* represent means ± S.E.

The role of inactivation gating was probed further using N588K and S620T attenuated inactivation mutants located in spatially distinct modules of the channel (see Fig. 1). Asn-588 is located in the S5-pore linker region, and the N588K mutation produces a +60- to +90-mV shift in voltage dependence of inactivation gating (41). Ser-620 is located on the pore helix, and S620T largely abolishes *I*_hERG_ inactivation (22). A concentration of Cavalli-2 that produced profound inhibition of WT *I*_hERG_ (300 nm), shown in Fig. 5*A*, inhibited N588K *I*_hERG_ by <50% (Fig. 5*B*) with an IC_50_ of 544 nm (CI, 445–664) and Hill coefficient (*n*_H_) of 0.97 (CI, 0.76–1.17) and produced only a small effect on S620T *I*_hERG_ (Fig. 5*C*) with an IC_50_ of 1916 nm (CI, 1434–2561) and *n*_H_ of 0.63 (CI, 0.47–0.79). Thus, attenuation or removal of channel inactivation had very significant effects on Cavalli-2 action, consistent with an important role for inactivation in optimally exposing the binding site(s) on the channel for the compound.

### Interactions of Cavalli-2 with canonical binding determinants in the inner cavity

**Figure 6.**
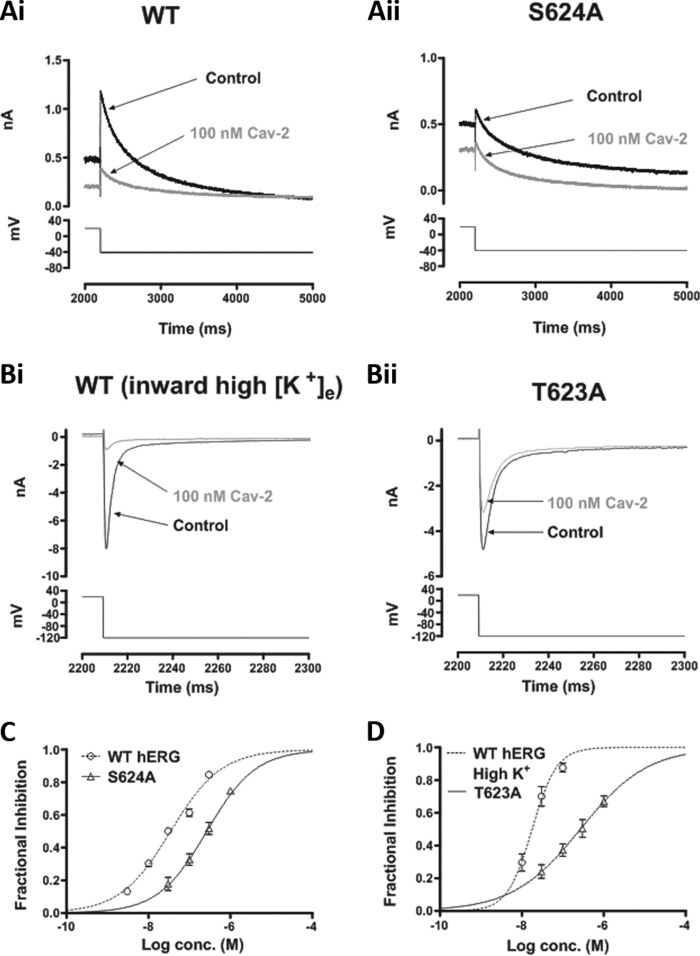
**Effect of pore helix mutations on *I*_hERG_ blockade by Cavalli-2.** Representative current traces from two pore helix mutants (*A*, *panel ii*, S624A; *B*, *panel ii*, T623A) before and after application of 100 nm Cavalli-2 (*Cav-2*) with their respective WT control current traces (*A*, *panel i*, and *B*, *panel i*) under appropriate recording conditions (see “Materials and methods”) are shown. *C*, concentration-response relationships for inhibition of S624A and WT *I*_hERG_ tails at −40 mV by Cavalli-2 (*n* ≥ 5 for each concentration of each curve). *D*, concentration-response relationships for inhibition of T623A and WT *I*_hERG_ tails by Cavalli-2 at −120 mV in high K^+^. *n* ≥ 5 for each concentration of each curve. *Error bars* represent means ± S.E.

High-affinity methanesulfonanilide *I*_hERG_ inhibitors have binding determinants within the hERG channel inner cavity that include residues at the base of the pore helix/selectivity filter and aromatic residues on the S6 helix (9, 15, 16). We investigated the effects of alanine mutagenesis of four of these: Thr-623, Ser-624, Tyr-652, and Phe-656. T623A and F656A typically exhibit low expression levels and are evaluated through the measurement of inward *I*_hERG_ tails at a negative voltage in high [K^+^]*_e_* (18–20, 33). This requires companion experiments on WT *I*_hERG_ under similar conditions. We determined an IC_50_ for inhibition of inward *I*_hERG_ in 94 mm [K^+^]*_e_* of 17.5 (CI, 13.8–22.1) nm and *n*_H_ of 0.99 (CI, 0.55–1.42). Fig. 6*A* compares the effects of 100 nm Cavalli-2 on S624A with its WT control, showing an attenuated effect for the mutant channel. Fig. 6*B* compares the effects of 100 nm Cavalli-2 on WT and T623A hERG at −120 mV, showing attenuated inhibition for the latter. Fig. 6*C* shows superimposed concentration-response relationships for WT and S624A *I*_hERG_; the IC_50_ for the mutant was 262 nm (CI, 209–328) with *n*_H_ of 0.72 (CI, 0.58–0.87), 7-fold that of its WT control. Fig. 6*D* shows superimposed concentration-response relationships for WT and T623A *I*_hERG_; the derived IC_50_ for the latter was 281 nm (CI, 196–403) with *n*_H_ of 0.54 (CI, 0.39–0.69), 16-fold that of its WT control.

**Figure 7.**
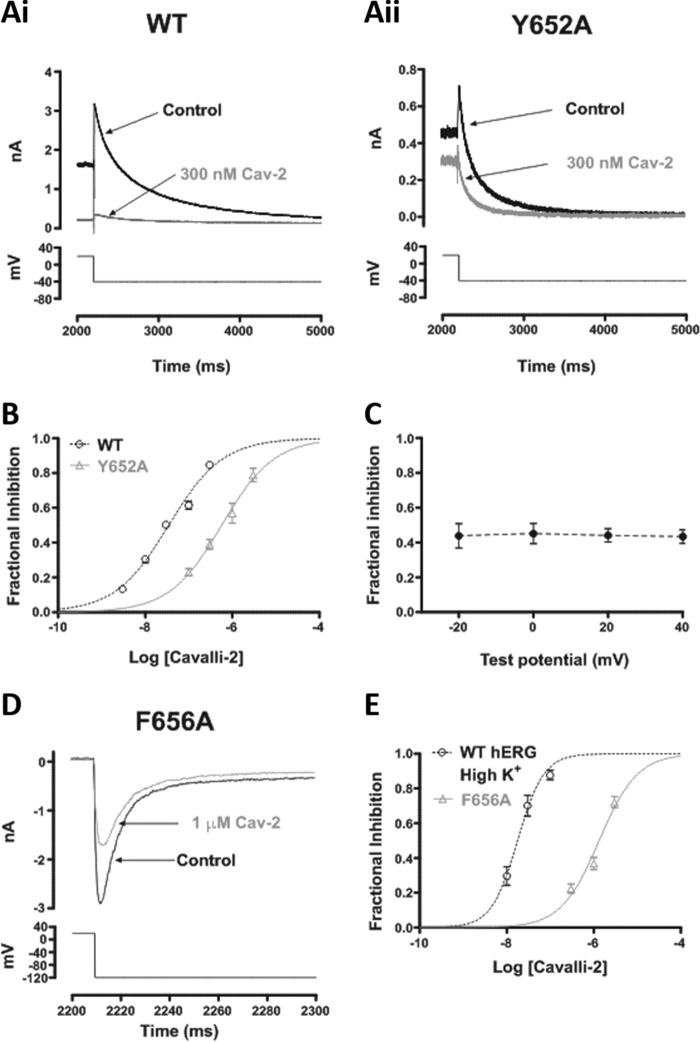
**Effect of S6 aromatic residue mutations on *I*_hERG_ by Cavalli-2.** Representative current traces from Y652A (*B*) before and after application of 300 nm Cavalli-2 (*Cav-2*) and its WT control (*A*) are shown. *C*, mean ± S.E. fractional block data for Y652A *I*_hERG_ tails following voltage commands to −20, 0, +20, and +40 mV. Data are from six cells. *D*, concentration-response relationships for inhibition of Y652A and WT *I*_hERG_ tails at −40 mV by Cavalli-2 (*n* ≥ 5 for each concentration of each curve). *E*, representative current traces from F656A at −120 mV in high K^+^ before and after application of 1 μm Cavalli-2. *F*, concentration-response relationships for inhibition of F656A and WT *I*_hERG_ tails at −120 mV in high K^+^. *n* ≥ 5 for each concentration of each curve. *Error bars* represent means ± S.E.

Fig. 7*A* compares effects of 300 nm Cavalli-2 on Y652A *I*_hERG_ (*panel ii*) with WT *I*_hERG_ (*panel i*) under similar recording conditions. Although this concentration produced a profound inhibition of the WT channel, it had only a modest inhibitory effect on Y652A *I*_hERG_. Fig. 7*B* shows corresponding concentration-response relationships with an IC_50_ for Y652A *I*_hERG_ of 594 nm (CI, 466–756) and *n*_H_ of 0.74 (CI, 0.58–0.90), 17-fold that of its WT control. The Tyr-652 residue has previously been reported to influence voltage dependence of inhibition for some compounds (13, 14, 42). We therefore compared Y652A *I*_hERG_ inhibition by 300 nm Cavalli-2 at 20-mV intervals from −20 to +40 mV. In contrast to the situation with the WT channel (Fig. 2*D*), there was no significant voltage dependence to the inhibitory effect of the compound on Y652A *I*_hERG_ tails (Fig. 7*D*; *p* > 0.05; one-way ANOVA, Bonferroni post hoc; *n* = 6). Fig. 7*E* shows the effect of 1 μm Cavalli-2 on F656A *I*_hERG_; this concentration reduced the current by ∼40%. Fig. 7*F* shows a concentration-response relationship for inhibition of F656A *I*_hERG_ with a derived IC_50_ of 1315 nm (CI, 1004–1725) and *n*_H_ of 0.93 (CI, 0.65–1.21) superimposed with that for WT *I*_hERG_ under similar conditions. This mutation had a profound effect on the inhibitory action of Cavalli-2: the derived IC_50_ was 75-fold that of its WT control.

**Figure 8.**
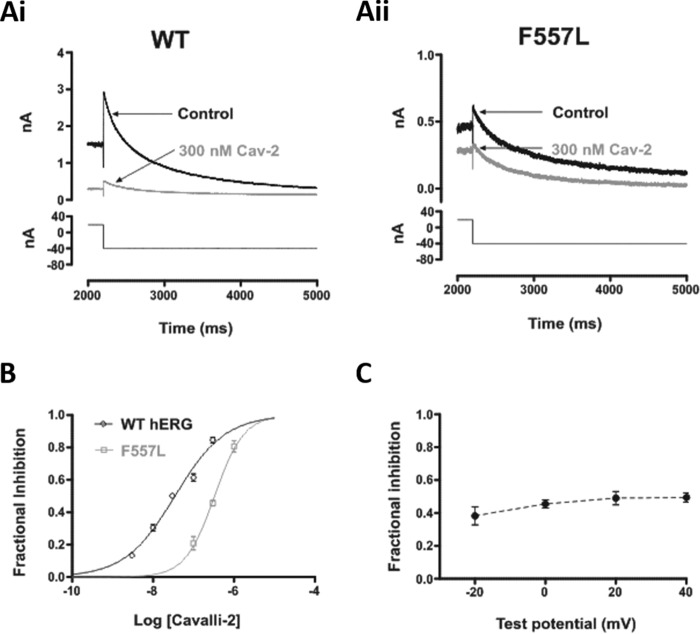
**Effect of S5 aromatic residue mutation on *I*_hERG_ by Cavalli-2.** Representative current traces from F557L (*A, panel ii*) before and after application of 300 nm Cavalli-2 (*Cav-2*) and its WT control (*A, panel i*) are shown. *B*, concentration-response relationships for inhibition of F557L and WT *I*_hERG_ tails at −40 mV by Cavalli-2 (*n* ≥ 5 for each concentration of each curve). *C*, mean ± S.E. fractional block data for F557L *I*_hERG_ tails following voltage commands to −20, 0, +20, and +40 mV. Data are from six cells. *Error bars* represent means ± S.E.

A recent report has suggested a novel binding determinant for hERG-blocking drugs, residue Phe-557 located on the channel's S5 helix (28). The F557L mutation was reported to decrease the inhibitory potency of a number of drugs, including the methanesulfonanilide dofetilide (28). We therefore investigated the effects of the F557L mutation on the action of Cavalli-2. Fig. 8*A* compares the effect of 300 nm Cavalli-2 on WT (*panel i*) and F557L (*panel ii*) *I*_hERG_, showing a reduced effect of the compound on the mutant channel. Fig. 8*B* shows superimposed concentration-response relationships for WT and F557L *I*_hERG_. The derived IC_50_ for inhibition of F557L *I*_hERG_ was 339 nm (CI, 293–392) with *n*_H_ of 1.2 (CI, 0.94–1.47), 10-fold that of its WT control. The mechanisms by which Phe-557 influences drug binding to hERG are not yet well elucidated but in principle could involve direct or indirect effects; in some homology models, this residue lies close to Tyr-652 (28), although in the cryo-EM structure of a hERG open pore construct (8) Phe-557 and Tyr-652 are not in direct contact. We further investigated the effects of the F557L mutation by comparing F557L *I*_hERG_ inhibition by 300 nm Cavalli-2 at 20-mV intervals from −20 to +40 mV. In contrast to the situation with the WT channel (Fig. 2*D*), there was no significant voltage dependence to the inhibitory effect of the compound on F557L *I*_hERG_ tails (Fig. 8*C*; *p* > 0.05; one-way ANOVA, Bonferroni post hoc; *n* = 6). Thus, similar to Tyr-652, Phe-557 influences the voltage dependence of *I*_hERG_ inhibition by Cavalli-2. Effects of all the mutations on *I*_hERG_-blocking potency of Cavalli-2 are summarized in Table 1.

**Table 1:** **Effect of pore helix, S5, and S6 mutations and mutations attenuating inactivation on *I*_hERG_ inhibition by Cavalli-2**

Channel	Voltage step	K^+^	IC_50_ (CI)	*n*_H_ (CI)	Potency reduction compared with its WT-control (fold WT IC_50_)
	*mV*	*m**m*	*n**m*		
WT-1	−40	4	35.6 (30.9–41.1)	0.69 (0.62–0.77)	
WT-2	−120	94	17.5 (13.8–22.1)	0.99 (0.55–1.42)	
N588K	−40	4	544 (445–664)	0.97 (0.76–1.17)	15.3
S620T	−40	4	1916 (1434–2561)	0.63 (0.47–0.79)	53.8
T623A	−120	94	281 (196–403)	0.54 (0.39–0.69)	16.1
S624A	−40	4	262 (209–328)	0.72 (0.58–0.87)	7.4
Y652A	−40	4	594 (466–756)	0.74 (0.58–0.90)	16.7
F656A	−120	94	1315 (1004–1725)	0.93 (0.65–1.21)	75.1
F557L	−40	4	339 (293–392)	1.20 (0.94–1.47)	9.5

### Computational docking

We searched for low-energy-score outputs for Cavalli-2 docked into the open pore structure of the recent cryo-EM structure (Fig. 1 and Ref. 8) using GOLD and Flexidock (29). We previously showed that IC_50_ values of positively charged hERG blockers correlate with the number of noncovalent interactions between drug and pore residues in optimized hERG pore models (29). The simple structure of Cavalli-2 combined with its strong hERG binding affinity suggests that each of the phenyl rings and the protonated nitrogen should contribute to binding. We also showed previously that GOLD docking scores broadly correlate with IC_50_ values (29). In analyzing docking data, we assessed the compatibility of docking outputs (poses) with the mutagenesis data, which provide information on residues that may interact with Cavalli-2. For example, the large attenuation of block in hERG F656A indicates that more than one Phe-656 side chain should interact with Cavalli-2. We also assessed GOLD docking outputs quantitatively using ChemPLP and ChemScore scoring (Table S1).

**Figure 9.**
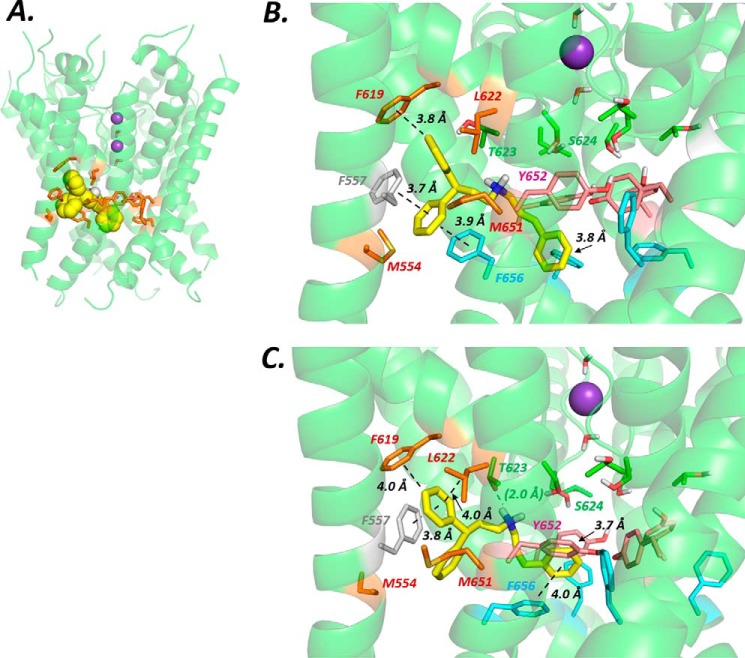
*A*, location of one of four equivalent hydrophobic pockets in the pore domain of the hERG cryo-EM structure with Cavalli-2 docked in the configuration shown in *B*. Amino acid side chains that comprise the pocket (*brown*) were allowed to rotate freely during docking runs to accommodate the drug. Potassium ions (*purple spheres*) in the 1 and 3 positions of the selectivity filter and waters (in positions 2 and 4) were added for docking runs. Cavalli-2 is represented as a space-filling *yellow* surface. *B*, low-energy-score pose for Cavalli-2 docked into the hERG pore with docking biased to promote occupation of a hydrophobic pocket. In this run, rotamers of two Phe-656 side chains adjacent to the pocket containing Cavalli-2 were selected to orient the side chain Cα–Cβ bond toward the pore and fixed during docking to allow Cavalli-2 to interact with more than one Phe-656 side chain. Annotations define noncovalent interactions between drug and amino acid side chains according to the criteria in Table 2 of Dempsey *et al*. (29); only interactions that satisfy these criteria are annotated. *C*, as in *B* but no side chain rotamers were fixed during docking. In all structure figures, the hERG pore amino acid side chains are colored as follows: Phe-557, *gray*; Met-554, Phe-619, Leu-622, and Met-651, *brown*; Thr-623 and Ser-624, *green*; Tyr-652, *pink*; and Phe-656, *blue*. Cavalli-2 is *yellow*.

Wang and Mackinnon (8) highlighted potential drug-binding “pockets” that lie beneath the selectivity filter and project outward from the K^+^ permeation pathway toward the S5 helix (Figs. 1 and 9). We docked Cavalli-2 into this part of the channel by biasing docking runs to include one of the pockets as the drug-binding site as indicated in Fig. 9 (stereo version in Fig. S4). The side chains of residues lining the pocket as well as the three Tyr-652 and Phe-656 residues nearest the selected pocket could rotate freely during docking to allow the drug to access the pocket and to optimize interactions with aromatic side chains, especially Phe-656. However, low-energy-score poses involving interactions with more than one Phe-656 side chain were not found.

In the cryo-EM open pore structure, the Phe-656 side chains adopt an unexpected orientation, projecting away from the central pore (Figs. 9 and 11*A* and Ref. 8). The distance between Phe-656 side chain phenyl groups in this configuration is large compared with distances expected from hERG pharmacophore models (*e.g.* Ref. 30), and Cavalli-2 could not simultaneously interact with “pocket” residues and with more than one Phe-656 side chain. By selecting an appropriate Phe side chain rotamer, Phe-656 side chains can be reoriented toward the hERG pore. However, this rotamer is poorly compatible with Phe side chains in a helical context due to unfavorable interactions with the *i* − 4 backbone carbonyl group (of Tyr-652) and, in the hERG structure, disruption of side chain packing at the interface of the S5 and S6 helices. Both GOLD and Flexidock reoriented the side chain back to the cryo-EM structure configuration during docking, allowing only a single Phe-656 interaction with Cavalli-2. Low-energy-score poses could be obtained with multiple (two) Phe-656 interactions by fixing the Cα–Cβ bond rotamer of Phe-656 residues adjacent to the pocket (and allowing rotation around the Cβ–Cγ bond of the Phe-656 side chain) to maintain the projection of these side chains toward the pore. A low-energy-score pose is shown in Figs. 9*B* and 11*B*. In this and similar low-energy-score poses, Cavalli-2 made aromatic π-π stacking interactions with two adjacent Phe-656 side chains and with Phe-557 and Phe-619 aromatic rings, but interactions with Tyr-652 were not favored.

Interestingly, rotation of a single Phe-656 side chain toward the pore cavity could be obtained in unconstrained docking runs in configurations where one of the Cavalli-2 phenyl rings replaced the Phe-656 side chain in its location packed between the S6 and S5 helices (Figs. 9*C* and 11*C*). In this pose, Cavalli-2 interacted with side chains of Phe-557, Phe-619, Thr-623, and Tyr-652 but only a single Phe-656 side chain (the side chain displaced into a pore-facing configuration).

**Figure 10.**
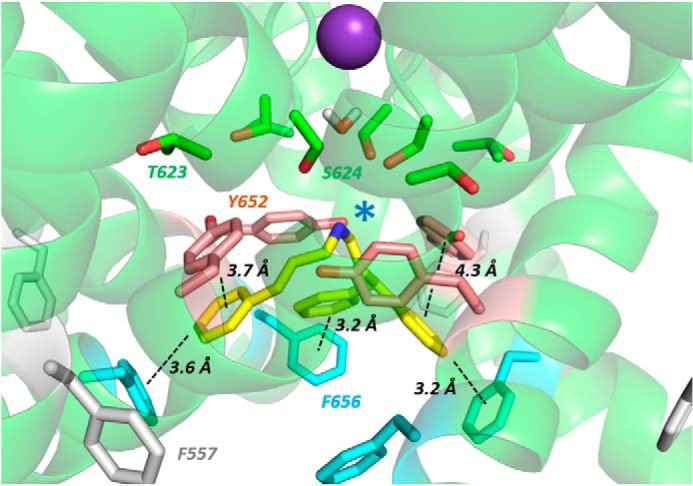
**Low-energy-score pose for Cavalli-2 docked into the MthK-based hERG pore model.** Annotations are as described in Fig. 9 legend. The *blue star* indicates the location of the protonated aliphatic amino group of Cavalli-2 near the internal binding site for a K^+^ ion where the C-terminal negative helix dipole charges from the four pore helices are focused. Amino acid side chain colors are as described in Fig. 9 legend.

We also docked Cavalli-2 into a hERG model based on the bacterial K^+^ channel MthK because this model produces low-energy-score poses that are consistent with experimental mutagenesis data for hERG blockers (19, 29) (see Fig. S2 for the sequence alignment). A representative low-energy-score pose is shown in Fig. 10. In this model, the phenyl rings of Cavalli-2 made multiple interactions with the S6 helix aromatic side chains, especially Phe-656. The protonated amino group lay near the internal K^+^-binding site where the negative electrostatic potential from the C-terminal pore helix dipole charges is focused (Fig. 10, *blue star*) so that all four functional groups of the drug made interactions with hERG. In these poses, Cavalli-2 could interact with Ser-624 (*e.g.* by direct or water-mediated hydrogen bonding with the protonated amino group) but not with the Phe-557 side chain. This type of pose for Cavalli-2 is consistent with a recent demonstration for several positively charged hERG inhibitors, using unnatural amino acid substitution, that neither Tyr-652 nor Phe-656 participate in cation-π interactions (43); *i.e.* the most likely binding contribution of the protonated amino group of high-affinity hERG blockers is via interaction at the internal K^+^-binding site, possibly involving H-bond interactions with Ser-624 (Fig. 10).

Comparison with the MthK model is also useful in estimating a minimal rotation of the S6 helix in the hERG cryo-EM structure that would accommodate projection of the Phe-656 side chains toward the pore in a configuration compatible with multiple interactions with high-affinity hERG blockers. A small clockwise rotation of the S6 helix by 20–30° in the hERG structure would position the Cα carbon of Phe-656 in a position on S6 relative to the pore axis that is equivalent to that for the Phe-656 Cα atoms on S6 in the MthK model. Similarly, in a hERG model built onto the closed pore structure of the highly homologous rat EAG cryo-EM structure (58), a relatively small clockwise rotation of the lower part of the S6 helix allows orientation of the Phe-656 side chain toward the K^+^ permeation pathway (Fig. S3).

**Figure 11.**
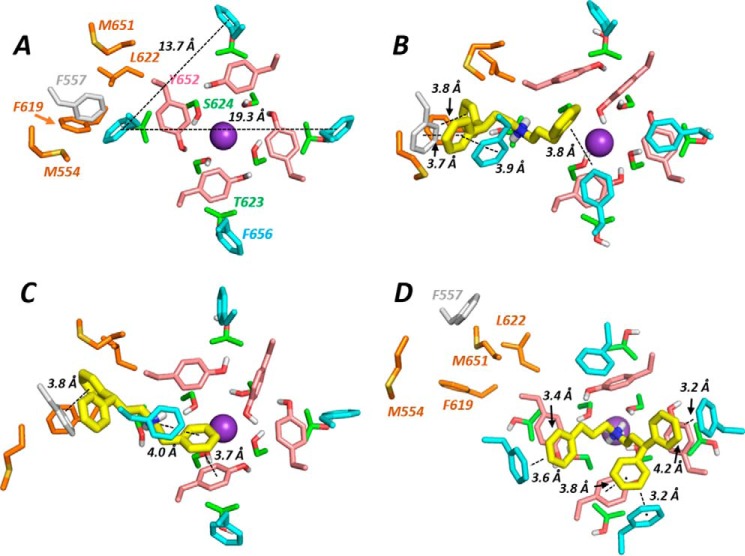
**Lowest-energy-score docked poses viewed from the cytoplasmic side of the channel pore.** *A*, the hERG construct cryo-EM structure (8) with distances between Phe-656 phenyl group centers marked. *B*, Cavalli-2 docked into hERG EM structure with selected Phe-656 side chains constrained to project toward the pore during docking; this is the same docking output as in Fig. 9*B. C*, as in *B* but with Phe-656 side chains unconstrained during docking as in Fig. 9*C. D*, unconstrained docking of Cavalli-2 into the hERG MthK-based model as in Fig. 10.

In summary, interaction of Cavalli-2 with potential binding pockets of the hERG cryo-EM structure resulted in limited interactions with Phe-656 side chains that are expected from mutagenesis data (Fig. 7 and Table 1) to dominate binding. Only if side chain rotamers were chosen to force Phe-656 orientations toward the channel pore could simultaneous interaction of Cavalli-2 with pocket residues and more than one Phe-656 side chain be obtained (Figs. 9*B* and 11*B*). The best docking scores using both ChemPLP and Goldscore scoring (Table S1) were obtained with a hERG pore model built on the MthK template in which Cavalli-2 made multiple interactions with Phe-656 side chains (Figs. 10 and 11*D*).

## Discussion

### Cavalli-2 interaction with WT hERG and inactivation-deficient mutants

Our IC_50_ value for Cavalli-2 in human embryonic kidney (HEK) cells at 37 °C (36 nm) is similar to the value (17 nm) determined in HEK cells at room temperature by Cavalli *et al.* (30), consistent with the compound acting as a high-affinity inhibitor of *I*_hERG_. The electrophysiology data in this study allow features of Cavalli-2 block to be defined in detail. The envelope-of-tails data of Fig. 3 indicate that Cavalli-2 is a gated-state inhibitor with little or no affinity for the closed channel state, and the steep increase in block over the rising phase of the *I*_hERG_ activation curve (Fig. 2*D*) suggests that channel activation is required for the drug to access its binding site. The δ value from the Woodhull analysis (0.2) is similar to that for other drugs that interact with residues within the hERG K^+^ permeation pathway (10, 36, 44) and indicates that Cavalli-2 binds at a site toward the interior of the membrane electric field. The three-step protocol of Fig. 4*D* indicates that Cavalli-2 binds to both the activated and inactivated states of hERG with a slight preference for the activated state as indicated by the slightly reduced block at voltages that promote inactivation (Fig. 4*E*). These features of Cavalli-2 block are compatible with those exhibited by drugs with more complex structures.

Cavalli-2 block of *I*_hERG_ nevertheless showed a clear reliance on the *process* of inactivation for maximal drug block as inhibition was significantly reduced for hERG mutants with attenuated inactivation. The reduction of drug block in these mutants is associated with attenuation of inactivation *per se* (rather than direct drug interactions with the mutated residues) because the attenuated inactivation N588K mutation resides in a helical structure in the hERG turret in the extracellular region of the hERG pore far from the drug-binding site (Fig. 1). As observed with other hERG blockers that depend strongly on inactivation competence for block (19, 22, 45), Cavalli-2 block was more strongly reduced for the pore helix S620T mutant, which has more profoundly attenuated inactivation. Considered alongside our WT hERG data, the reduction in block in the inactivation-attenuated mutants suggests that a conformational state of the drug-binding site that is associated with the ability of hERG to inactivate is required for high-affinity block. This is in accordance with the results of Chen *et al.* (24) who showed that inactivation competence is associated with conformational changes that bring aromatic side chains of Tyr-652 and Phe-656 into an optimal configuration for drug binding and that this is likely to involve rotation of the S6 helices that contain these residues (24).

### Alanine mutagenesis

Cavalli-2 drug block was attenuated by alanine substitution mutation of Thr-623 and Ser-624 at the C-terminal end of the pore helix and Tyr-652 and Phe-656 on the S6 helix (see Fig. 1); these are all residues whose alanine substitution attenuates block by a variety of high-affinity hERG blockers (4, 9, 12, 15, 17), indicating that these molecules share a similar binding site in the hERG pore. Cavalli-2 block is particularly susceptible to the F656A mutation (75-fold attenuation of block), suggesting that the drug interacts with more than one Phe-656 side chain. *I*_hERG_ block was attenuated for Y652A hERG by 17-fold, and this is compatible with interaction of a Cavalli-2 phenyl ring with one or more Tyr-652 side chains. The loss of the voltage dependence of Cavalli-2 block in Y652A (Fig. 7*C*) is not simply attributable to altered voltage-dependent activation of mutant compared with WT channels as the voltage dependence of activation of Y652A hERG has been shown to be similar to that of the WT channel (9). Rather, it implicates Tyr-652 as critical for voltage-dependent block through voltage-dependent changes in the configuration of the Tyr-652 side chain that optimizes its interactions with drugs. This effect (loss of voltage dependence of block in Tyr-652 mutants) has been observed previously for other drugs, including vesnarinone, quinidine, moxifloxacin, cisapride, and chloroquine (13, 42, 46).

The F557L mutation has recently been reported to attenuate drug block by a number of “classical” hERG blockers with the suggestion that this residue may provide an additional binding determinant for hERG pore blockers (28). Cavalli-2 block was moderately reduced in hERG F557L (by 10-fold), and as observed for a number of drugs by Saxena *et al.* (28), the extent of block attenuation in F557L (10-fold) was similar to that found for Y652A (17-fold). Interestingly, as with the Y652A mutant, the voltage dependence of Cavalli-2 block was nearly abolished in F557L (Fig. 8). The voltage dependence of activation of F557L hERG is only slightly (−5.5 mV) leftward shifted compared with that of the WT channel (47), and this is insufficient to account for the large effect of the mutation on voltage dependence of *I*_hERG_ inhibition by Cavalli-2. The broadly similar effects on drug block of Y652A and F557L in this study and previously (28), together with the loss of voltage dependence of block in each mutant, suggest that these residues may interact together in promoting block by drugs that require optimal configurations of the Tyr-652 side chain. These side chains do not interact directly in the hERG cryo-EM structure but are part of a small cluster of aromatic side chains involving Phe-557, Tyr-652, and Phe-656 (Fig. 1 and Ref. 8). Direct voltage responsiveness of Tyr side chains is mediated by the dipole moment of the Tyr phenolic hydroxyl group (48), which is absent in the Phe side chain; loss of voltage sensitivity in F577A is therefore likely to be mediated via effects on voltage sensitivity of other voltage-responsive side chains such as Tyr-652.

### Structural interpretations

To what extent can our mutagenesis data be interpreted within a structural model for high-affinity drug block, particularly in the context of the new hERG structure in which pockets below the selectivity filter were proposed to form part of the drug-binding site? The low nanomolar IC_50_ value of the structurally simple Cavalli-2 molecule indicates that binding interactions should involve contributions from each of its three phenyl groups and single protonated amino group in accordance with the pharmacophore model on which the structure of Cavalli-2 was based (30). Thus, Cavalli-2 should be a useful probe of the spatial configuration of drug-binding groups, especially the aromatic side chains of Tyr-652 and Phe-656; substantial interactions with multiple (at least two) Phe-656 side chains seem to be required to understand the large attenuations of block seen with several high-affinity blockers, including flecainide (33), Cavalli-2 (this study), E-4031, and cisapride (27) in hERG F656A-overexpressing mammalian cells and MK-499, cisapride (9), chloroquine (14), and clofilium (17) in hERG F656A-overexpressing oocytes.

Contrary to these expectations, docking of Cavalli-2 into the hERG cryo-EM structure with the binding site selected to promote interaction with one of the hydrophobic pockets fails to find low-energy-score poses in which Cavalli-2 can interact with more than one Phe-656 side chain. This results from the orientation of the side chains of Phe-656 that project away from the pore axis toward the S5 helix in the cryo-EM structure (Figs. 1 and 11*A*). Phe-656 aromatic side chains are too far apart in this configuration to allow interaction of two phenyl rings on Cavalli-2 with two separate Phe-656 side chains while making interactions within a hydrophobic pocket. Only when Phe-656 side chain rotamers were constrained to reorient side chains toward the pore could multiple interactions between Cavalli-2 phenyl groups and multiple Phe-656 side chains be obtained (Fig. 9*B*). However, these rotamers are not favored due to Phe-656 side chain clashes with the Tyr-652 backbone carbonyl group and the loss of side chain packing between the S5 and S6 helices. If unconstrained during docking, the side chains reorient back to the configuration found in the EM structure. It is unlikely therefore that the apparent discrepancy between high dependence on Phe-656 and the limited interactions of Cavalli-2 with Phe-656 side chains in the cryo-EM structure can be resolved by postulating reorientation of Phe-656 side chain(s) during drug binding; more substantial conformational changes from the cryo-EM structure are required for Cavalli-2 to interact with more than one Phe-656 side chain as described below.

One explanation for an apparent mismatch between the mutagenesis data and the hERG EM structure is that some of the large effect of Phe-656 mutations on attenuation of drug block in Cavalli-2 and other high-affinity hERG blockers is allosteric; *i.e.* loss of side chain packing between the S5 and S6 helices resulting from replacement of a bulky phenyl group with a methyl group in F656A results in a collapse of optimal configurations of drug-binding groups. It has been observed, for example, that retention of high binding affinity for MK-499 and cisapride can be retained if Phe-656 is replaced by bulky aromatic or aliphatic side chains, which should be more suitable than Ala for filling the space occupied by the Phe-656 side chain phenyl rings in the hERG EM structure (49). However, this interpretation is poorly compatible with the retention of unperturbed block by cisapride, E-4031, and terfenadine in hERG tandem dimers in which two diagonally opposite Phe-656 side chains are replaced with Ala (27); this shows that replacement of at least two Phe-656 side chains with Ala does not perturb the high-affinity binding site for these blockers. The inability to find docking outputs involving Phe-656 for a number of classical hERG blockers in the new cryo-EM open pore structure has led to the suggestion that the role of Phe-656 is entirely to provide optimal orientations of Tyr-652 for drug binding (50). However, this interpretation is incompatible with the finding for a number of hERG blockers, including Cavalli-2, that the effect of F656A on attenuating hERG block is greater or, in the case of some drugs (*e.g.* propafenone (10)), much greater than the effect of Y652A.

Some of the disparity between mutagenesis and docking data may be reconciled by considering conformational changes involving reorientation of S6 aromatic side chains associated with the voltage dependence of hERG block and with hERG inactivation. The association of high-affinity hERG block with inactivation has previously been suggested to involve reconfiguration of Tyr-652 and Phe-656 aromatic side chains such that interactions with hERG-blocking drugs in the channel pore are optimized. Chen *et al.* (24) showed that this likely involves rotation of the S6 helix to reorient the aromatic side chains toward the pore; drug block susceptibility in the noninactivating hERG homolog EAG was conferred by moving S6 aromatic side chains by one residue to change their projection from the S6 helix with respect to the channel K^+^ permeation path. For both Tyr-481 (equivalent to Tyr-652 in hERG) and Phe-485 (equivalent to Phe-656 in hERG), cisapride sensitivity was conferred by shifting the aromatic amino acid one residue toward the C terminus, a movement equivalent to a clockwise rotation of the S6 helix by 100° (24). A smaller clockwise rotation of the S6 helix (*e.g.* by 30–40°) would move the side chain of Phe-656 in the hERG EM structure to a pore-facing configuration that would allow interaction of Cavalli-2 with more than one Phe-656 side chain in a configuration similar to that for the MthK-based hERG pore model (Figs. 10 and S3). Because Cavalli-2 block shows approximately equivalent preference for an open and open-inactivated state (the open state has slightly higher affinity; Fig. 4, *D* and *E*), such a configuration resulting from reorientation of S6 is not the inactivated state *per se* but is only optimized in channels that are able to inactivate as described also by Chen *et al.* (24).

Further clues to an explanation for the disparities between our experimental data for Cavalli-2 and docking to the open state cryo-EM structure come from cryo-EM data on an inactivation-attenuated S631A mutant that were published alongside that of the WT template (8). Aside from a subtle repositioning within the selectivity filter of S631A, the two structures are very similar with no differences in the orientation of S6 helices or configuration of S6 aromatic side chains. It seems unlikely therefore that the cryo-EM structure of the WT hERG construct is the inactivated state as suggested previously (8); an alternative explanation is that the open channel structure captured for the WT hERG construct in the cryo-EM structure is that of a low-affinity open state (*e.g.* as proposed by Imai *et al.* (27) from analysis of the kinetics of cisapride binding to WT hERG and hERG mutants). In this state, the configurations of binding residues are not optimal for interaction with compounds such as Cavalli-2. A small clockwise rotation of the S6 helix in the hERG WT cryo-EM structure would bring the S6 aromatic side chains, in particular Phe-656, into a configuration more compatible with experimental data on inactivation-dependent blockers (*e.g.* see Fig. 10).

### Conclusions

Despite its simple structure, Cavalli-2 shares features of high-affinity hERG block with many structurally complex high-affinity blockers (*e.g.* MK-499, E-4031, terfenadine, dofetilide, and haloperidol), including a requirement for inactivation competence in hERG channels and a strong dependence on Phe-656 for block. Docking using the new open pore hERG structure finds low-energy-score poses in which Cavalli-2 extends into hydrophobic pockets that were proposed to account for the unique promiscuity of hERG toward a variety of structurally diverse high-affinity blockers. However, in these states, Cavalli-2 does not make interactions with the multiple Phe-656 side chains that seem to be required for the strong dependence of Phe-656 on high-affinity block. The close structural similarity between the WT cryo-EM structure and a copublished inactivation-attenuated S631A hERG open pore structure, together with the evidence that high-affinity binding to inactivation-competent hERG channels may be associated with rotation of S6 helices to reorient Tyr-652 and especially Phe-656 aromatic side chains into optimal positions or high-affinity drug block (24), suggests that both WT and inactivation-attenuated S631A cryo-EM structures may correspond to a low-affinity pore configuration that is not optimal for high-affinity block.

## Materials and methods

### WT and mutant hERG channels

A HEK293 cell line stably expressing WT hERG channels was kindly donated by Prof. Craig January (51). The pore helix (T623A and S624A) and S6 helix (F656A) alanine mutants, attenuated-inactivation mutants (N588K and S620T), and S5 mutant F557L were all generated and used as described previously (3, 18, 41, 52). The HEK293 cell line stably expressing the hERG S6 helix mutant Y652A was used in this study as described previously (52).

### Maintenance of mammalian cell lines and cell transfection

HEK293 cells stably or transiently expressing hERG constructs were maintained as described previously (18, 19, 52). Cells were plated in 40-mm Petri dishes at least 48 h before transfection and incubated at 37 °C with 5% CO_2_. Cells were transfected either with Lipofectamine^TM^ 2000 (Invitrogen) or Lipofectamine LTX (Invitrogen) following the manufacturer's instructions. The amount of hERG construct DNA that was transfected varied between 0.1 and 1.0 μg depending on the level of functional expression. 0.15 μg of CD8 was cotransfected as a transfection marker, and successfully transfected cells were identified using Dynabeads® (Invitrogen). Cells were plated on sterilized glass shards in 40-mm Petri dishes and incubated at 37 °C (5% CO_2_) at least 24 h before electrophysiological recording; this was to allow time for cell recovery and hERG construct expression.

### Mutagenesis

The F557L hERG mutation was generated using the Quik Change® site-directed mutagenesis kit (Stratagene, La Jolla, CA). In brief, a pair of complementary oligonucleotide primers containing the mutation (forward primer sequence, 5′CTCATGTGCACCTTAGCGCTCATCG-3′; reverse primer sequence, 5′CGATGAGCGCTAAGGTGCACATGAG-3′; synthesized by Sigma-Genosys, Haverhill, UK) was used in a PCR (95 °C for 1 min, 60 °C for 1 min, and 68 °C for 16 min for 18 cycles) by using hERG in a modified pcDNA3.0 vector as a DNA template. A DpnI digest of the PCR mixture was then performed for 1 h at 37 °C. Competent DH5α *Escherichia coli* (Invitrogen) were transformed using standard procedures. The mutation was confirmed by sequencing of the entire ORF (Eurofins MWG Operon, Ebersberg, Germany).

### Solutions, electrophysiology, and experimental protocol

For *I*_hERG_ recordings, glass shards containing plated cells were placed in a recording chamber mounted on an inverted microscope (Nikon Diaphot). The cells were superfused continuously with Tyrode's solution containing 140 mm NaCl, 4 mm KCl, 2.5 mm CaCl_2_, 1 mm MgCl_2_, 10 mm glucose, 5 mm HEPES (titrated to pH 7.4 with NaOH). As described previously (18, 19), a modified “high-K^+^” version of this solution (containing 94 mm KCl and 50 mm NaCl) was used to obtain recordable currents from comparatively poorly expressed hERG mutants (T623A and F656A). Patch pipettes (Schott number 8250 glass, A-M Systems Inc.) were pulled (Narishige, PP 830) and polished (Narishige, MF 83) to final resistance values between 2 and 4 megaohms. The patch pipettes were filled with intracellular solutio, containing 130 mm KCl, 1 mm MgCl_2_, 5 mm EGTA, 5 mm MgATP, and 10 mm HEPES (titrated to pH 7.2 with KOH). Pipette resistance compensation was between 70 and 80%. Cavalli-2 (30), which was synthesized by Ascent Scientific (Abcam Ltd.), was dissolved in DMSO to produce 100 μm, 1 mm, and 10 mm stock solutions. Stock solutions were diluted either in standard or high-K^+^ Tyrode's solution as appropriate to obtain final concentrations stated under “Results.” Solutions were preheated to 37 ± 1 °C and applied to single cells using a homemade, multibarreled superfusion system, enabling rapid superfusate exchange (<1 s) (53). Thus, measurements of *I*_hERG_ were made at 37 ± 1 °C as described previously (18, 19, 52, 54, 55).

All *I*_hERG_ recordings were made using an Axopatch 200B amplifier (Axon Instruments, now Molecular Devices) and a CV203BU head stage. Data were acquired using a Digidata 1320 interface (Axon Instruments, now Molecular Devices). Data digitization rates were between 10 and 25 kHz during all voltage protocols, and an appropriate bandwidth between 2 and 10 kHz was set on the amplifier. For WT hERG and most of the mutant channels studied, activating voltage commands to +20 mV were used with tail currents observed at either +40 (for most mutants) or −120 mV (for T623A and F656A) (18–20, 33). The level of block of WT and mutant *I*_hERG_ by Cavalli-2 was attained by repetitive protocol application for 5 min, and fractional inhibition of *I*_hERG_ tails was measured. Data for each mutant were compared with WT *I*_hERG_ under comparable conditions (18, 19, 33, 52).

### Data analysis and statistics

Data analysis was performed using Clampfit 10.3 (Axon Instruments, now Molecular Devices), Prism versions 4.03 and 5.03, and Excel 2013. Data are presented as the mean ± S.E. or as mean with ±95% CI. Equations used to fit particular data sets are given in the supporting information. Statistical comparisons were made using paired or unpaired two-tailed *t* tests, Wilcoxon matched-pairs signed-rank test, or one-way ANOVA followed by a Bonferroni post-test as appropriate. Details of the statistical test used to evaluate significance for results of particular experiments are given alongside *p* values under “Results” or in the relevant table or figure legend. *p* values of < 0.05 were taken as statistically significant.

The fractional block (FB) of hERG tail currents by the range of drug concentrations studied was determined using the following equation,

(1) $$\text{FB}=1-\frac{I_{\text{hERG}-\text{drug}}}{I_{\text{hERG}-\text{control}}}$$

where *I*_hERG-drug_ and *I*_hERG-control_ are the amplitude of tail currents, respectively, in the presence and absence of a defined concentration of Cavalli-2; the ratio of these two values represented the fraction of unblocked *I*_hERG_ (see Fig. 1*B*). Cavalli-2 block reached a steady state within 5 min; therefore, rundown correction was not required.

Concentration-response relationships were constructed by plotting mean fractional block of hERG tail currents against concentration of Cavalli-2 and then fitting the experimental data with a standard Hill equation, yielding the IC_50_ and the *n*_H_ values,

(2) $$y=1/\left(1+10^{\wedge }\left(\left(\log\;{\text{IC}}_{50}-x\right)\times n_{\text{H}}\right)\right)$$

where *x* is the concentration expressed as a logarithm, *y* is the fractional block of *I*_hERG_ at a given concentration, and IC_50_ and *n*_H_ are as defined above.

The voltage dependence of *I*_hERG_ activation was established by using the step protocol reported in Fig. 1*D*. The normalized tail current amplitude was plotted against the command potential of the previous depolarizing step, and the experimental data points were fitted with a Boltzmann function of the following form,

(3) $$I=I_{\max}/\left(1+\exp\left(V_{0.5}-x/\text{Slope}\right)\right)$$

where *I* is the tail current elicited after the test voltage *x*, *I*_max_ is the maximal current recorded, *V*_0.5_ is the voltage that elicits half-maximal activation, and Slope is the slope factor of the curve. The same equation was used to simulate the *I-V* curves shown in Fig. 1*D*.

The fraction of the electrical transmembrane field sensed by a single positive charge at the binding site of hERG was calculated to further probe the voltage dependence of hERG blockade by Cavalli-2. *K_D_* values for Cavalli-2 inhibition at +60 mV and a reference voltage of 0 mV were estimated and substituted into the following equation,

(4) $$K_{D\;+\;60\;\text{mV}}=K_{D\;0\;\text{mV}}\times \exp\left(1-\frac{z\delta FV}{RT}\right)$$

where *K_D_*_+60__mV_ and *K_D_*_0__mV_ represent half-maximal blocking concentrations at +60 and 0 mV, respectively; *V* is membrane test potential (+60 mV in this instance); and *z*, *R*, *F*, and *T* have their usual meanings (see Refs. 35 and 56).

The voltage dependence of inactivation was studied using the three-step protocol shown in Fig. 3, *inset*. The *I*_hERG_ transients at the beginning of the third step were analyzed as described previously (41, 57). The normalized current was plotted against the test voltage during the 2-ms second repolarization step; experimental data were then fitted with the following Boltzmann equation,

(5) $$I/I_{\max}=1-\left(1+\exp\left[V_{0.5}-V_{m}\right]/k\right)$$

where *I* is the current amplitude at the beginning of the third step, *I*_max_ is the maximal recorded current after the 2-ms repolarization step to varying voltages *V_m_*, *V*_0.5_ is the potential that elicits half-maximal inactivation, and *k* is the slope factor for the relationship.

To obtain the time course of inactivation at +40 mV, the decay of the resurgent current at the beginning of the third step after a 2-ms repolarization step to −120 mV was fitted with a single exponential equation of the following form,

(6) y=A×exp⁡(−x/τ) + C

where *y* is *I*_hERG_ recorded at time *x*, τ is the time constant of the decay of the transient current, *A* represents the total fitted current, and *C* is the residual current after the decline of the resurgent current.

### Molecular modeling and docking

The following structures and models were used for computational docking and homology model construction. Docking was largely done using the recent cryo-EM open pore structure of hERG (Protein Data Bank code 5VA1) (8). A homology model of a hERG closed pore state was built onto the rat EAG closed pore cryo-EM structure (Protein Data Bank code 5K7L) (58) using MODELLER 9.17 (59) with PROCHECK (60) to assess model quality. We also used a homology model of an open pore hERG state that was built onto the X-ray crystal structure of MthK (Protein Data Bank code 1LNQ) (61) because this model provides a consistent match between experimental Ala-scanning data and computational docking for several hERG blockers (19, 20, 29). Following publication of the rat EAG (58) and hERG atomic resolution structures (8), we realigned the S5 helix of this model; the alignment of the pore helix, selectivity filter, and S6 helix is the same as described previously (the full alignment is shown in Fig. S2).

Computational docking was done using two independent docking methods, GOLD and Flexidock as described previously (20, 29). Free side chain flexibility was normally allowed during docking for selected residues incorporating the chain A hydrophobic pocket (Phe-557, Phe-619, Leu-622, Met-651, and Tyr-652), Tyr-652 of chains B and D, and three Phe-656 side chains (chains A, B, and D) to maximize the binding of Cavalli-2 within or near the pocket. The binding pocket was centered above the β-carbon of the chain A Tyr-652 residue, and a radius of 12 or 15 Å was selected to allow Cavalli-2 to sample configurational space within the pocket and surrounding parts of the pore. Due to the large number of rotamers sampled during docking, 300,000 generations of the genetic algorithm were used; all GOLD runs were performed twice to obtain outputs scored with both ChemPLP and ChemScore functions (GOLD version 5.6; Cambridge Crystallographic Data Centre, Cambridge, UK). Low-energy-score poses were inspected from sets of 100 docking repeats. In some cases, the center of the pore cavity below the selectivity filter at a level between the Tyr-652 and Phe-656 side chains was chosen as the binding site, and in these runs all Tyr-652 and Phe-656 side chains were allowed free side chain rotamer sampling. Other variations, including fixing Phe-656 side chain rotamers to promote interaction with Cavalli-2 in the pore, were used as described under “Results.” Flexidock docking was done as described previously (20, 29) using full rotamer flexibility of side chains in the Cavalli-2–binding region selected. Structural figures were made using PyMOL version 1.4 (Schroedinger, LLC, New York, NY).

## Author contributions

M. V. H. and C. E. D. data curation; M. V. H., J. C. H., and C. E. D. formal analysis; M. V. H., Y. Z., A. E. H., C. D., J. C. H., and C. E. D. investigation; M. V. H., J. C. H., and C. E. D. writing-original draft; M. V. H., Y. Z., A. E. H., C. D., J. C. H., and C. E. D. writing-review and editing; Y. Z., A. E. H., C. D., J. C. H., and C. E. D. resources; J. C. H. and C. E. D. conceptualization; J. C. H. and C. E. D. supervision; J. C. H. and C. E. D. funding acquisition; J. C. H. and C. E. D. methodology; J. C. H. and C. E. D. project administration.

## Supplementary Material

Supporting Information

## Footnotes

4 The abbreviations used are:

hERG: human ether-à-go-go–related gene
*I*: current
CI: confidence interval
EAG: ether-à-go-go
ANOVA: analysis of variance
AP: action potential.
