# Supplementary material for: Structural implications of hERG K+ channel block by a high-affinity minimally structured blocker
Source: J Biol Chem. 2018 Mar 15;293(18):7040–57. doi: 10.1074/jbc.RA117.000363 (PMC5936838; doi:10.1074/jbc.RA117.000363)
Supplement: Supporting Information [file supp_293_18_7040__index.html]

Structural implications of hERG K+ channel block by a high-affinity minimally structured blocker — hERG channel block by Cavalli-2 — Supporting Information 

# Structural implications of hERG K+ channel block by a high-affinity minimally structured blocker

## Supporting Information

- Supporting Information for Helliwell et al - A pdf documenting containing the supporting Information figures and tables referred to in the main text and to be published with the main article
