# Supplementary material for: Structural implications of hERG K+ channel block by a high-affinity minimally structured blocker
Source: J Biol Chem. 2018 Mar 15;293(18):7040–57. doi: 10.1074/jbc.RA117.000363 (PMC5936838; doi:10.1074/jbc.RA117.000363)
Supplement: Supporting Information [file supp_RA117.000363_133023_1_supp_69031_p3qdj7.pdf]

Structural implications of hERG K^+ channel block by a high affinity minimally-structured blocker

Matthew V. Helliwell, Yihong Zhang, Aziza El Harchi, Chunyun Du, Jules C. Hancox and Christopher E. Dempsey

SUPPORTING INFORMATION

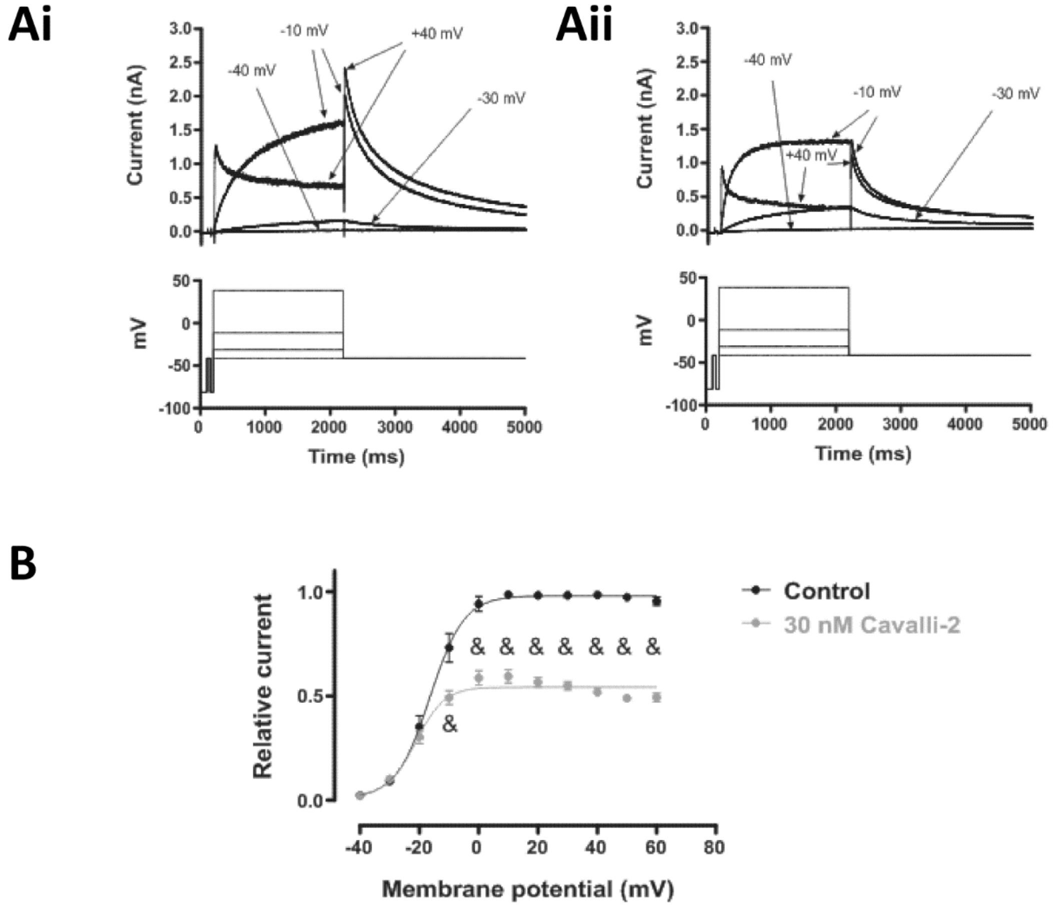

Figure S1. A) Representative current traces of I_{hERG} elicited by the step protocol illustrated in the lower panels in Control (i) and in the presence of 30 nM Cavalli-2 (ii); for clarity, selected currents at 4 test voltages are shown while the full protocol spanned from -40 to +60 mV in 10 mV increments. Peak I_{hERG} tails at -40 mV following each depolarising command were measured relative to that elicited by the initial brief 50 ms step from -80 mV to -40 mV.

B) Normalised I-V relationship for I_{hERG} tails in Control (black) and in presence of 30 nM Cavalli-2 (grey). Peak tail currents in both conditions were normalized to the maximal tail current amplitude recorded in Control (n=5, & $p<0.01$ 2-Way ANOVA with Bonferroni post hoc test). The experimental points were fitted with equation 3 of the main text. Control $V_{0.5}$ = -16.4 ± 0.8 mV, Slope 5.7 ± 0.6 and 30 nM Cavalli-2 $V_{0.5}$ = -21.1 ± 1.3 mV, Slope 4.8 ± 1.1. $\Delta V_{0.5}$ = -4.68 mV, (n=5).

A.

```
              --------- S5 ----------
MthK 19:      PATRILLLVLAVIIYGTAGFHFIEGESWT
hERG 549:     VLFLLMCTFALIAHWLACIWYAIEGESWT

              -pore helix- SF       ------------ S6 -------------
MthK 48:      VSLYWTFVTIATVGYGDYSPSTPLGMYFTVTLIVLGIGTFAVAVERLLEFL   :98
hERG 613:     TALYFTFSSLTSVGFGNVSPNTNSEKIFSICVMLIGSLMYASIFGNVSAII   :663
```

B.

S5	pore helix	hERG EM structure	model
L564	T618	4.0 Å	4.6 Å
A565	L615	3.8 Å	4.8 Å
W568	A614	4.6 Å	4.8 Å

Figure S2. A) Alignment of hERG and MthK S5 helix, pore helix, selectivity filter (SF; yellow highlight) and S6 helix sequences used to construct the MthK-based hERG pore homology model using PDB:1LNQ as a structural template. The MthK loop sequence (underlined) connecting the top of S5 and the pore helix was built into the hERG pore homology model to replace the turret region between S5 and the pore helix in hERG (see Figure 1 of main text). The structure of the pore from residues 613 to 663 in the hERG homology model is the same as the structure in the MthK-based hERG pore homology model described previously (Dempsey et al., 2014).

B) The hERG and MthK S5 sequences align poorly. The alignment was chosen which oriented F557 (red in the hERG sequence in A) towards the S6 helix at an equivalent height with respect to Y652 on S6 as in the hERG construct cryo-EM structure (Wang and MacKinnon, 2017), and which best reproduced the interactions between side chains at the top of S5 and in the pore helix as determined by distances (shown in B) between side chain β-carbons where these helices pack together in the cryo-EM structure.

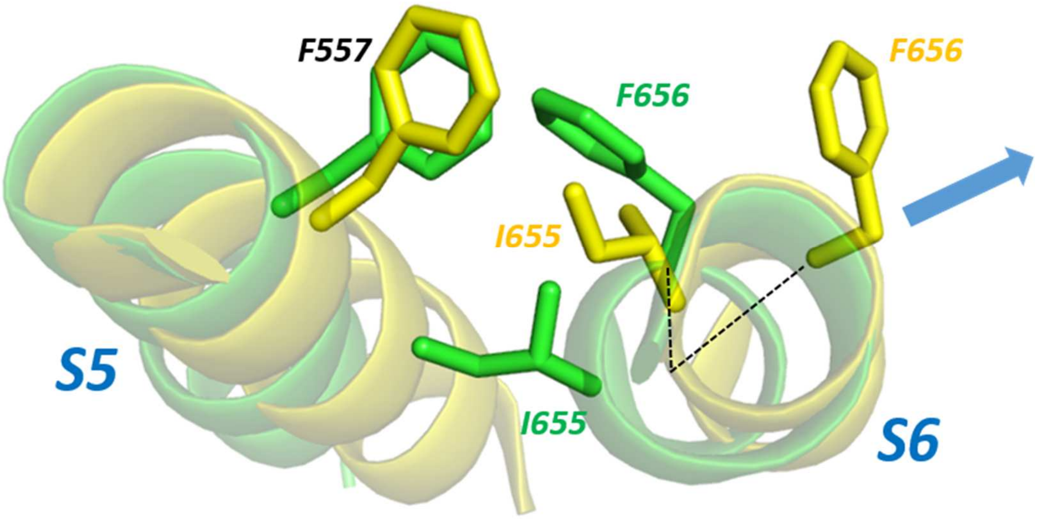

Figure S3. Superposition of a segment of a single subunit of the hERG cryo-EM structure (Wang and MacKinnon, 2017) (green) containing parts of the S5 and S6 helices that include F557 (S5) and I655 and F656 (S6), with the equivalent segment of a hERG closed pore model built onto the rat EAG cryo-EM structure (Whicher and MacKinnon, 2017) (yellow). The part of the S6 helix containing I655 and F656 is oriented in the closed state model such that the side chain of F656 projects towards the K^+ permeation pathway (the blue arrow indicates the direction of the pore center). In the open state the F656 side chain projects away from the K^+ permeation pathway (see Figure 1 of main paper). The movement of F656 from open to closed pore structures is equivalent to a clockwise rotation of the S6 helix by around 45 ^{o} and brings the side chain of I655 in the closed state model into an equivalent position as F656 in the open state structure.

Table S1. GOLD docking scores for Cavalli-2

Structure	***optimised for interaction***	***representative pose***	***CHMPLP***	***Chemscore***	***(ΔG_{app})***
hERG construct EM	"pocket" fixed F656 rot[1]	Fig. 9b; Fig 11b	98.0	39.0	(-43.6)
hERG construct EM	"pocket" free F656 rot[2]	Fig 9c; Fig 11c	99.0	42.0	(-45.0)
MthK model	no bias	Fig. 10; Fig 11d	105.5	47.5	(-50.2)

----

[1] Binding site (10 Å radius) centred above β-carbon of Y652 chain A. F656 side chains of chains B and D Cα-Cβ rotamer chosen to project side chains towards pore to allow multiple F656 interactions.

[2] As above but free F656 rotamer sampling allowed.

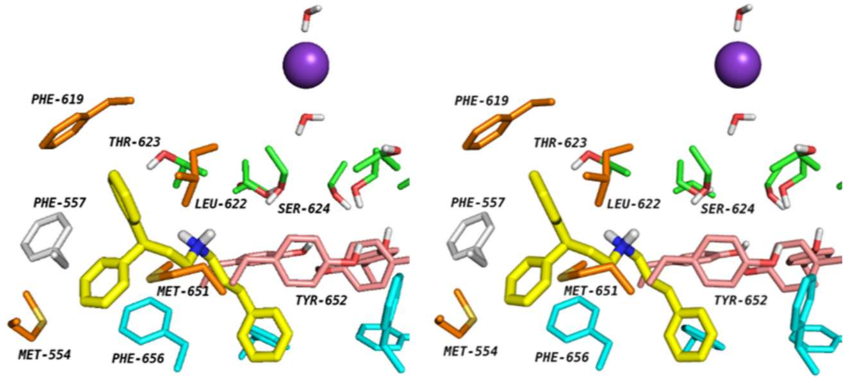

A. Cavalli-2 docked into hERG cryo EM structure with side chain rotamers of F656 fixed to project side chains towards pore. Simplified stereo version of Figure 9b of main paper.

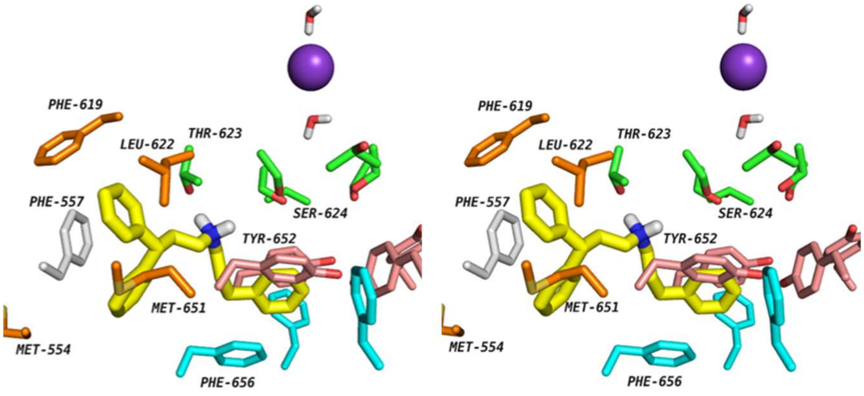

B. Cavalli-2 docked into hERG cryo EM structure with free rotation of F656 side chains. Simplified stereo version of Figure 9c of main paper.

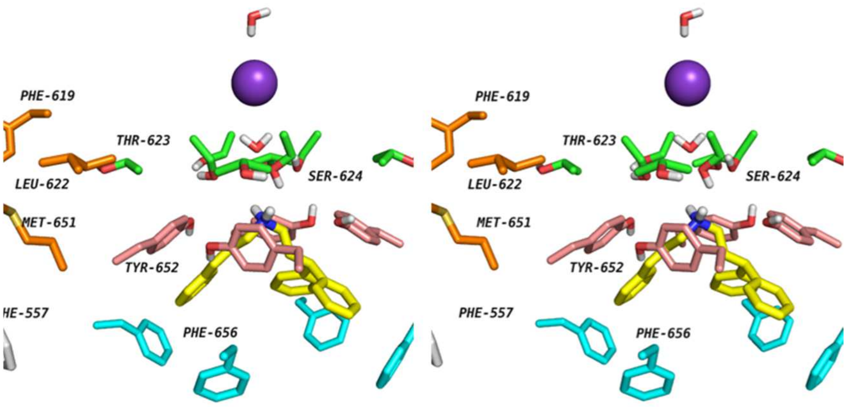

C. Cavalli-2 docked into MthK-based hERG pore model with free rotation of F656 side chains. Simplified stereo version of Figure 10 of main paper.

Figure S4. Stereo images of docking outputs described in the text.

References:

Dempsey, C. E., Wright, D., Colenso, C. K., Sessions, R. B. and Hancox, J. C. (2014) Assessing hERG pore models as templates for drug docking using published experimental constraints: the inactivated state in the context of drug block. J. Chem. Inf. Model. 54, 601–612.

Wang, W. and Mackinnon, R. (2017) Cryo-EM Structure of the Open Human ether-à-go-go-Related K^+ Channel hERG. Cell 169, 422–424.

Whicher, J. R. and MacKinnon, R. (2017) Structure of the voltage-gated K^+ channel Eag1 reveals an alternative voltage sensing mechanism. Science **353**, 664–669.
